# Fusing Multimodal and Anatomical Volumes of Interest Features Using Convolutional Auto-Encoder and Convolutional Neural Networks for Alzheimer’s Disease Diagnosis

**DOI:** 10.3389/fnagi.2022.812870

**Published:** 2022-04-28

**Authors:** Mohammed Abdelaziz, Tianfu Wang, Ahmed Elazab

**Affiliations:** ^1^National-Regional Key Technology Engineering Laboratory for Medical Ultrasound, Guangdong Key Laboratory for Biomedical Measurements and Ultrasound Imaging, School of Biomedical Engineering, Health Science Center, Shenzhen University, Shenzhen, China; ^2^Department of Communications and Electronics, Delta Higher Institute for Engineering and Technology (DHIET), Mansoura, Egypt; ^3^Computer Science Department, Misr Higher Institute of Commerce and Computers, Mansoura, Egypt

**Keywords:** Alzheimer’s disease, multimodal images, convolutional auto-encoder, convolutional neural networks, anatomical volumes of interest

## Abstract

Alzheimer’s disease (AD) is an age-related disease that affects a large proportion of the elderly. Currently, the neuroimaging techniques [e.g., magnetic resonance imaging (MRI) and positron emission tomography (PET)] are promising modalities for AD diagnosis. Since not all brain regions are affected by AD, a common technique is to study some region-of-interests (ROIs) that are believed to be closely related to AD. Conventional methods used ROIs, identified by the handcrafted features through Automated Anatomical Labeling (AAL) atlas rather than utilizing the original images which may induce missing informative features. In addition, they learned their framework based on the discriminative patches instead of full images for AD diagnosis in multistage learning scheme. In this paper, we integrate the original image features from MRI and PET with their ROIs features in one learning process. Furthermore, we use the ROIs features for forcing the network to focus on the regions that is highly related to AD and hence, the performance of the AD diagnosis can be improved. Specifically, we first obtain the ROIs features from the AAL, then we register every ROI with its corresponding region of the original image to get a synthetic image for each modality of every subject. Then, we employ the convolutional auto-encoder network for learning the synthetic image features and the convolutional neural network (CNN) for learning the original image features. Meanwhile, we concatenate the features from both networks after each convolution layer. Finally, the highly learned features from the MRI and PET are concatenated for brain disease classification. Experiments are carried out on the ADNI datasets including ADNI-1 and ADNI-2 to evaluate our method performance. Our method demonstrates a higher performance in brain disease classification than the recent studies.

## Introduction

Alzheimer’s disease (AD) is the main cause of dementia that normally worsens over time (Khachaturian, 1985; Kucmanski et al., 2016; Anter et al., 2019). The memory loss and cognitive impairment are the most common symptoms of AD. The mild cognitive impairment (MCI) is an intermediate stage between healthy people and AD that can be classified into two subgroups; stable MCI (sMCI) and progressive MCI (pMCI) (Chen et al., 2016). As there is no treatment to revert AD, the early detection of AD is the only way to prevent patients from losing their memory and other cognitive abilities from deterioration (Hao et al., 2020). Therefore, researchers utilized the neuroimaging data [e.g., magnetic resonance imaging (MRI) and positron emission tomography (PET)] to identify AD due to their abilities to provide a complementary structural and functional information of human brain (Zhu et al., 2017; Bi et al., 2020; Folego et al., 2020).

Various machine learning methods have been proposed in literature for AD diagnosis from single and multimodal images. Zu et al. (2016) jointly selected a subset of relevant features from multiple modalities *via* a group sparsity regularizer and fused the selected features for AD diagnosis. Zhang et al. (2018) utilized the neuroimaging and genetic data for learning a multi-layer multi-view classification technique for AD diagnosis. Zhang et al. (2011) proposed a framework that used a kernel combination method for brain disease classification. Liu et al. (2012) combined multiple individual classifiers such that each classifier utilized different subsets of local patches. Liu et al. (2014a) developed a multitask feature selection method to preserve inter-modality relationship by imposing a constraint and employed support vector machine (SVM) to combine the significant features for AD diagnosis. Min et al. (2014) registered each subject with multiple atlases and calculated the correlation among them to select the relevant features then used the SVM for classification. Zhang et al. (2012) developed a multimodal multi-task learning that selected the significant features from each modality. They also used SVM to fuse the features for brain disease classification and regression. Liu et al. (2014b) developed a hierarchical ensemble classification method that gradually transformed the high-dimensional imaging into a compact representation by constructing multi-level classifiers. Tong et al. (2017) calculated the pairwise similarity matrix from multi-modal data then fused the similarities from each modality for classification. Zhou et al. (2019a) developed a latent representation learning framework that used all the available samples then projected the latent representations to the label space for classification.

Recently, various deep learning techniques have been also proposed in literature for AD diagnosis. Feng et al. (2019) designed a technique based on convolutional neural network (CNN) and fully stacked bidirectional long short-term memory for learning the neuroimaging data (MRI and PET) for AD diagnosis. In Abdelaziz et al. (2021), we designed a framework for classification and regression using neuroimaging and genetic data using CNN. Zhou et al. (2019b) developed a framework to discriminate patients with AD from healthy subjects using deep neural network in three stages. Liu et al. (2018b) developed a CNN model based on discriminative anatomical landmarks from MRI data for joint classification and regression. Suk et al. (2014) developed a deep learning framework that utilized a restricted Boltzmann machine for computer-aided AD/MCI diagnosis. In another work, Cheng et al. (2017) combined various learned features from local brain images using 3D-CNNs for classification. Similarly, Liu et al. (2018a) fused the learned multi-level and multi-modality features using cascaded CNNs model for improving the performance of AD. Also, Basaia et al. (2019) utilized the 3D T1-weighted images for predicting the individual diagnosis of AD and discriminating sMCI from pMCI using CNN.

Although the above-mentioned techniques achieved good performance, they still have some limitations. First, they only used either the handcrafted features or the original image. Second, they used the relevant patches instead of full image for classification. Specifically, they firstly selected the relevant discriminative patches relevant to AD. Then, the relevant patches are used for classification. Third, they either used MRI or PET for brain disease classification. Fourth, most methods adopted multistage learning scheme to learn from the multi-modal data. However, this learning scheme requires extensive processing and memory resources.

To address the above limitations, we develop a novel technique that takes the advantages of learning from both original neuroimaging features and their brain region-of-interests (ROIs) features. In addition, we use the ROIs features for forcing the network toward the regions that are highly related to AD. Specifically, we first convert the 116 ROIs features from the Automated Anatomical Labeling (AAL) atlas into the image space by registering every ROI with its corresponding region of the original image. Hence, for every subject, we have the original image and the synthetic image. Afterward, we train the original images using a CNN while train the synthetic images using a convolutional auto-encoder. Note that, we separately train the CNN and the convolutional auto-encoder for every modality. Meanwhile, we concatenate the features from CNN and convolutional auto-encoder after each convolution layer for each modality. Finally, the highly learned features from both modalities are concatenated for classification.

The objectives of this work are as follows. First, we aim to develop a method that uses multimodal neuroimaging data and boost attention to the highly related AD regions. Second, integrate the original neuroimaging features with their ROIs features in one framework. Meanwhile, we force the deep learning network to focus on the regions that are highly related to AD. Third, develop a method that converts the ROIs features to image space in order to utilize it as 3D image in deep learning framework. Finally, develop a deep learning model that utilizes the multimodal data in only one stage.

The remainder of this work is organized as follows. The neuroimaging dataset and its preprocessing are presented in section “Materials and Methods.” Furthermore, the methodology is introduced in section “Methodology.” The experimental results are given in section “Results and Discussions.” Lastly, conclusions and future work are summarized in section “Conclusions and Future Work.”

## Materials and Methods

### Subjects

In this study, we employed the public database of Alzheimer’s Disease Neuroimaging Initiative (ADNI)^1^ that has ADNI-1 and ADNI-2 phases for evaluating the proposed method. Table 1 presents the number of subjects in ADNI-1 and ADNI-2 datasets used in our study. Furthermore, we assess the efficiency of our method by using the common subjects between MRI and PET such that the number of the subjects is 959 including; 264 NC, 273 sMCI, 204 pMCI, and 218 AD.

**TABLE 1:** Number of subjects utilized in our work.

Dataset	Modality	NC	sMCI	pMCI	AD	Total
ADNI-1	MRI	213	211	159	180	763
	PET	97	121	75	91	384
ADNI-2	MRI	167	152	129	127	575
	PET	167	152	129	127	575
	Common	264	273	204	218	959

### Data Preprocessing

**FIGURE 1:**
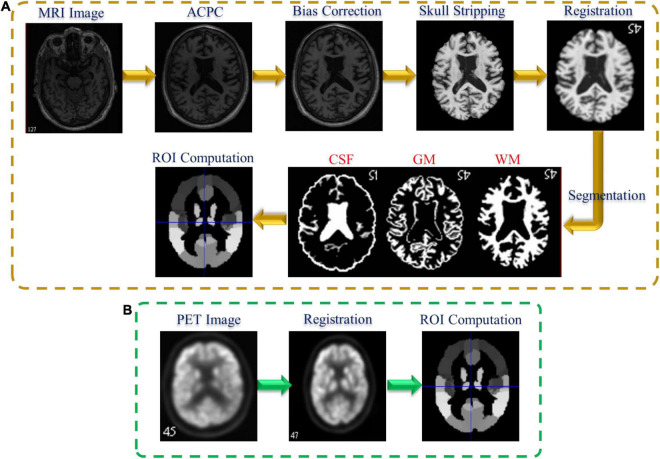
Neuroimaging data preprocessing pipeline: **(A)** MRI and **(B)** PET.

Figure 1 shows the preprocessing pipeline of the neuroimaging data. We perform the preprocessing of the neuroimaging data including MRI and PET as previously described by Zhou et al. (2019b). For the MRI preprocessing (Figure 1A), we first employ the MIPAV program for correcting the anterior commissure-posterior commissure (AC–PC). Then, N3 algorithm was applied to correct the bias field of the processed AC-PC images. Afterward, we perform the brain extraction using the skull-stripping technique in Min et al. (2014) followed by removing the cerebellum. Furthermore, we register the skull-stripped image with Montreal Neurological Institute template (Lancaster et al., 2000).

Following that, the three brain tissues [white matter (WM), gray matter (GM), and cerebrospinal fluid (CSF)] are extracted by employing the FAST algorithm in the FSL package (Zhang et al., 2001). Finally, to calculate the GM feature for each ROI in each subject, we register the segmented GM tissue with the atlas (Tzourio-Mazoyer et al., 2002) and normalize the corresponding GM volume to the total intracranial volume. On the other hand, Figure 1B shows the PET preprocessing pipeline. Firstly, the affine registration is employed to register each PET data with their corresponding T1 MR images. Then, we compute the PET ROIs features by averaging the intensity of each ROI.

## Methodology

### Overview of the Proposed Method

**FIGURE 2:**
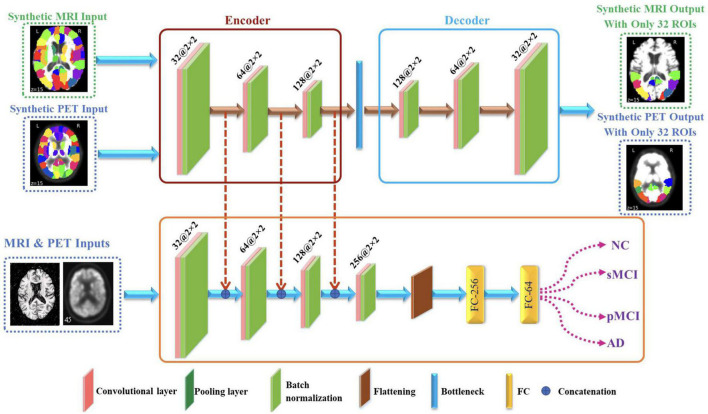
Proposed framework that utilizes convolutional auto-encoder and CNN for brain disease classification.

**FIGURE 3:**
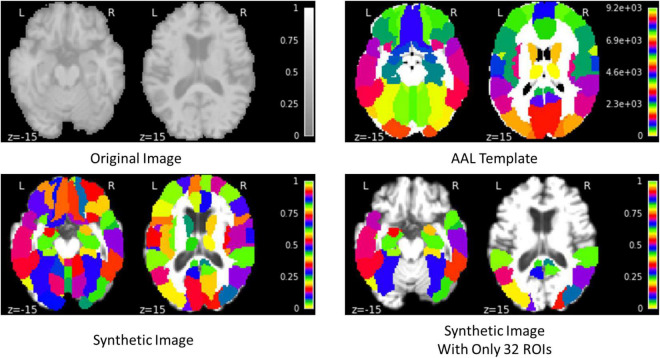
Example of generating the synthetic MRI image.

**FIGURE 4:**
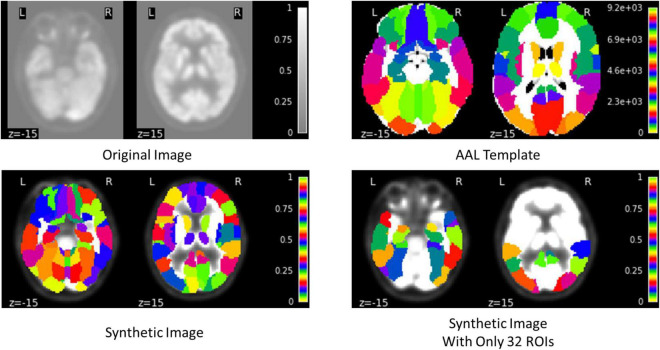
Example of generating the synthetic PET image.

The proposed framework for AD diagnosis using multimodality neuroimaging data is shown in Figure 2. In our framework, we employ the CNN to learn original features from MRI and PET and the convolutional auto-encoder to learn the 116 ROIs features in one framework. However, they cannot be used directly without converting the ROIs features to an image space. Thus, we convert the ROIs to image space with the aid of AAL by registering every ROI with its corresponding region of the original image as shown in Figures 3, 4. Hence, we create the synthetic images for MRI and PET modalities. Furthermore, we generate the synthetic MRI and PET images with only 32 ROIs features by registering only the regions in Table 2 as these regions are highly related to AD (Sun et al., 2009). Then, we develop a deep learning model that learns the synthetic images by convolutional auto-encoder and the original neuroimaging data by using CNN. Note that, we separately train the CNN and the convolutional auto-encoder for every modality. Meanwhile, we concatenate the features generated from each modality with those from the convolutional encoder after each convolution layer to force the network to focus on the selected regions related to AD. Finally, the highly features from MRI and PET are concatenated for brain disease classification.

**TABLE 2:** Highly AAL-related ROIs to AD.

Index	Name	Index	Name
35	Cingulum_Post_L	59	Parietal_Sup_L
36	Cingulum_Post_R	60	Parietal_Sup_R
37	Hippocampus_L	61	Parietal_Inf_L
38	Hippocampus_R	62	Parietal_Inf_R
39	ParaHippocampal_L	67	Precuneus_L
40	ParaHippocampal_R	68	Precuneus_R
41	Amygdala_L	81	Temporal_Sup_L
42	Amygdala_R	82	Temporal_Sup_R
49	Occipital_Sup_L	83	Temporal_Pole_Sup_L
50	Occipital_Sup_R	84	Temporal_Pole_Sup_R
51	Occipital_Mid_L	85	Temporal_Mid_L
52	Occipital_Mid_R	86	Temporal_Mid_R
53	Occipital_Inf_L	87	Temporal_Pole_Mid_L
54	Occipital_Inf_R	88	Temporal_Pole_Mid_R
55	Fusiform_L	89	Temporal_Inf_L
56	Fusiform_R	90	Temporal_Inf_R

### Convolutional Auto-Encoder

The convolutional auto-encoder is a type of auto-encoder designed specifically for image and multidimensional data processing. It extends the basic structure of the simple auto-encoder by replacing the completely linked layers to convolutional, down-sampling, and up-sampling layers (Guo et al., 2017). In literature, convolutional auto-encoder has been widely used in many different tasks such image de-noising (Ahmed et al., 2021), anomaly detection (Kolberg et al., 2021), and deep clustering (Snover et al., 2021).

Generally, the conventional auto-encoder is used to extract a latent feature representation of the input data without the need of labels and it comprises two main components; encoder *f*(*x*) and decoder*g*(*x*) (Wang et al., 2021). The encoder uses a mapping function to generate a latent features representation of the input data then the latent representation is used to reconstruct the input image using the decoder network. The applied loss function between the encoder input *x* and decoder output $\hat{x}=g\,\left(f\,\left(x\right)\right)$ is the mean squared errors (MSE) as follow:


(1) $$m\,i\,n\,\frac{1}{N}\,\sum_{i}\left(x_{i}-g\,\left(f\,\left(x_{i}\right)\right)\right),$$

where the total number of inputs is *N* and the latent representation output at the *Z*-layer are calculated for the *i*-th subject as:


(2) $$z_{i}=f\,\left(x_{i}\right)\to z_{i}\in {\mathbb{R}}^{Z}.$$

In this work, we use convolutional auto-encoder such that the input data is the synthetic images generated by registering every ROI with its corresponding region of the original image. On the other hand, the decoder uses the latent feature representation of the input data to reconstruct the synthetic images with only 32 ROIs highly related to AD as given in Table 2 (Sun et al., 2009). We reconstruct these regions to increase the attention to these regions and hence, increase the performance of disease classification.

In the proposed architecture, we use three convolutional layers for each synthetic image such that the rectified linear unit (ReLU) activation function and batch normalization are used after each convolutional layer. Meanwhile, after each convolution layer, the convoluted output is concatenated with the convoluted output generated from original images from CNN. Then, the decoder process is the same as encoder process but in reverse order for extracting the synthetic images with regions highly related to AD.

### Convolutional Neural Networks

The CNN is one of the most common deep learning techniques used for extracting the high learned features from the input data (LeCun et al., 1989). CNN has been frequently utilized in many different tasks such a face recognition, breast cancer diagnosis, AD diagnosis, and brain tumor detection (Díaz-Pernas et al., 2021; Naveen and Sivakumar, 2021; Zhang et al., 2021). It includes three main layers, namely convolutional, pooling, and fully connected (FC) layer (Bailer et al., 2018). The convolution layer is made up of various convolution kernels that are used to compute various input feature representations. In addition, the max-pooling is used for down sampling the convoluted output and hence it reduces the features dimensionality. Finally, the FC layer is used to equip the network with classification capabilities.

In our CNN model, we use three convolutional layers for each original image such that the ReLU and batch normalization are used after each convolutional layer. Meanwhile, the convoluted output is concatenated with the convoluted output generated from synthetic images. Then, the flattened layer is employed independently in each of the neuroimaging data to flatten the last convoluted output. Finally, the Softmax activation function is employed for identifying the disease. Note that, we utilize dropout layer in order to avoid the potential overfitting. Furthermore, the highly learned features from MRI and PET are concatenated and went through series of FC for classification.

## Results and Discussion

### Experimental Settings

In our study, we apply a different configuration of our method to show its efficiency in three different binary tasks (AD vs. NC, MCI vs. NC, and pMCI vs. sMCI). In addition, we compare the proposed method with many state-of-the-art machine learning and deep learning studies.

At the beginning, we randomly initialized the network with mean equal to 0 and standard deviation (SD) equal to 1. In addition, the binary cross-entropy and MSE were employed as loss functions for the CNN and convolutional auto-encoder, respectively. Furthermore, the optimizer was set to Adam, the number of epochs was set to 100, the batch size was set to 30, the learning rate was set to 10^–4^, and *k* was equal to 10 for *k*-fold cross-validation. Note that, we employ 10 independent experiments and average all the results with mean and SD.

We assess the performance of the proposed method using different evaluation measures including; accuracy (ACC), sensitivity (SEN), specificity (SPE), precision (PRE), and F1 score (F1). These measures are defined as follow:


(3) $$A\,c\,c=\frac{T\,P+T\,N}{T\,P+F\,N+T\,N+F\,P},$$

(4) $$S\,e\,n=\frac{T\,P}{T\,P+F\,N},$$

(5) $$S\,p\,e=\frac{T\,N}{T\,N+F\,P},$$

(6) $$F\,1=\frac{2\,T\,P}{2\,T\,P+F\,N+F\,P},$$

where TP, TN, FP, and FN are the number of true positives, true negatives, false positives, and false negatives, respectively.

### Comparison Methods

We verify the effectiveness the performance of the proposed method by comparing it to different machine learning methods including; Liu et al. (2014b), Tong et al. (2017), and Zhou et al. (2019a). Also, we compare it with different deep learning studies including; Suk et al. (2014), Cheng et al. (2017), Liu et al. (2018a; 2018b), Basaia et al. (2019), Feng et al. (2019), Zhou et al. (2019b), and Abdelaziz et al. (2021).

### Effects of Different Configuration of Our Method

**FIGURE 5:**
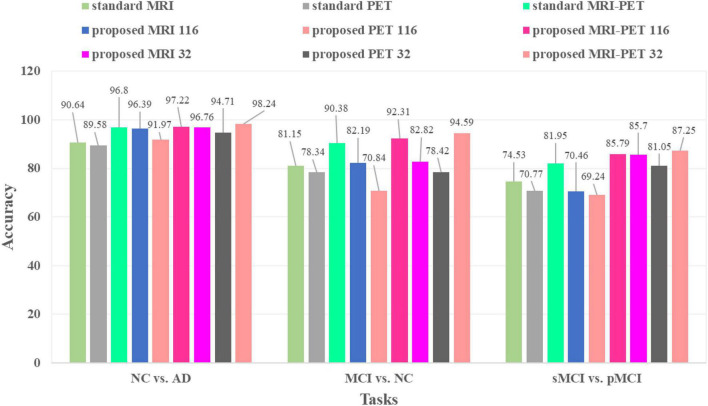
Classification accuracies comparison between the standard and the proposed method in three different tasks.

Table 3 and Figure 5 compare between different configuration of the standard and the proposed method. It is clear that, involving synthetic images not only increases the performance of the single modality but also the combination of modalities. Specifically, using only the MRI modality (standard MRI) achieves a classification accuracy of 90.64, 81.15, and 74.53% for NC vs. AD, MCI vs. NC, and pMCI vs. sMCI, respectively. While only using PET modality (standard PET) achieves 89.58, 78.34, and 70.77% for NC vs. AD, MCI vs. NC, and pMCI vs. sMCI, respectively. However, combining MRI and PET (standard MRI-PET) improves the accuracy and achieves 96.80, 90.38, and 81.95% for NC vs. AD, MCI vs. NC, and pMCI vs. sMCI, respectively.

**TABLE 3:** Classification comparison between the standard and the proposed method in three different binary disease classification tasks (%).

Tasks	Method	ACC	SEN	SPE	PRE	F1
NC vs. AD	Standard MRI	90.64 ± 2.89	87.84 ± 5.38	94.04 ± 2.42	94.74 ± 1.98	91.07 ± 3.04
	Standard PET	89.58 ± 1.39	90.00 ± 2.08	89.08 ± 2.93	90.95 ± 2.15	90.44 ± 1.25
	Standard MRI-PET	96.80 ± 0.63	97.50 ± 1.03	95.96 ± 1.64	96.72 ± 1.28	97.10 ± 0.57
	Proposed MRI 116	96.39 ± 2.94	98.90 ± 3.34	93.35 ± 6.68	95.02 ± 4.86	96.81 ± 2.57
	Proposed PET 116	91.97 ± 3.55	91.10 ± 8.65	93.03 ± 9.55	94.91 ± 6.50	92.47 ± 3.39
	Proposed MRI-PET 116	97.22 ± 2.74	97.92 ± 4.29	96.38 ± 4.48	97.17 ± 3.42	97.46 ± 2.55
	Proposed MRI 32	96.76 ± 2.48	99.96 ± 0.12	92.89 ± 5.47	94.61 ± 4.07	97.17 ± 2.15
	Proposed PET 32	94.71 ± 4.30	97.95 ± 6.47	90.78 ± 8.94	93.32 ± 6.36	95.30 ± 3.95
	Proposed MRI-PET 32	98.24 ± 3.03	98.82 ± 3.19	97.52 ± 6.15	98.19 ± 4.40	98.43 ± 2.66
MCI vs. NC	Standard MRI	81.15 ± 2.32	52.31 ± 7.69	97.11 ± 1.12	91.11 ± 2.59	66.10 ± 6.05
	Standard PET	78.34 ± 1.42	53.82 ± 4.71	91.91 ± 1.62	78.77 ± 1.62	63.81 ± 3.37
	Standard MRI-PET	90.38 ± 2.21	77.76 ± 5.64	97.36 ± 1.51	94.28 ± 2.94	85.12 ± 3.77
	Proposed MRI 116	82.19 ± 3.68	70.61 ± 20.35	88.59 ± 12.22	83.74 ± 16.15	72.83 ± 7.85
	Proposed PET 116	70.84 ± 7.98	35.64 ± 24.26	90.31 ± 18.37	82.10 ± 21.00	42.58 ± 21.15
	Proposed MRI-PET 116	92.31 ± 2.87	91.63 ± 9.83	92.68 ± 7.40	89.28 ± 10.16	89.50 ± 3.29
	Proposed MRI 32	82.82 ± 4.45	59.05 ± 13.81	95.97 ± 8.48	93.50 ± 13.72	70.47 ± 8.24
	Proposed PET 32	78.42 ± 8.38	46.10 ± 15.18	96.31 ± 10.88	93.69 ± 17.41	59.57 ± 15.33
	Proposed MRI-PET 32	94.59 ± 4.50	90.26 ± 8.82	96.98 ± 4.86	94.95 ± 8.13	92.19 ± 6.36
pMCI vs. sMCI	Standard MRI	74.53 ± 2.83	88.17 ± 14.02	56.27 ± 15.37	73.79 ± 4.61	79.44 ± 4.54
	Standard PET	70.77 ± 2.34	91.46 ± 2.26	43.09 ± 6.82	68.36 ± 2.36	78.20 ± 1.33
	Standard MRI-PET	81.95 ± 5.91	83.15 ± 17.53	80.34 ± 11.72	80.34 ± 11.72	83.18 ± 7.97
	Proposed MRI 116	70.46 ± 5.58	81.72 ± 20.15	55.39 ± 26.33	73.80 ± 10.20	75.11 ± 7.49
	Proposed PET 116	69.24 ± 7.85	73.11 ± 23.19	64.07 ± 26.14	76.32 ± 11.15	71.60 ± 11.16
	Proposed MRI-PET 116	85.79 ± 4.89	86.45 ± 12.13	84.90 ± 13.04	89.72 ± 7.76	87.18 ± 5.04
	Proposed MRI 32	85.70 ± 6.03	90.00 ± 14.65	79.95 ± 18.15	87.85 ± 9.10	87.47 ± 6.17
	Proposed PET 32	81.05 ± 7.47	79.16 ± 19.51	83.58 ± 14.89	88.50 ± 8.76	81.64 ± 9.77
	Proposed MRI-PET 32	87.25 ± 5.68	94.25 ± 8.65	77.89 ± 16.18	86.33 ± 9.14	89.49 ± 4.52

On the other hand, the synthetic MRI with 116 ROIs features (proposed MRI 116) achieves 96.39, 82.19, and 70.46% for NC vs. AD, MCI vs. NC, and pMCI vs. sMCI, respectively. Similarly, the synthetic PET with 116 ROIs features (proposed PET 116) achieves 91.97, 70.84, and 69.24% for NC vs. AD, MCI vs. NC, and pMCI vs. sMCI, respectively. Fusing the synthetic MRI and PET with 116 ROIs features (proposed MRI-PET 116) achieves 97.22, 92.31, and 85.79% for NC vs. AD, MCI vs. NC, and pMCI vs. sMCI, respectively. It is clear that the synthetic image (MRI and/or PET) can effectively improve the classification accuracy of the disease.

Furthermore, the synthetic MRI with 32 ROIs features (proposed MRI 32) achieves a classification accuracy of 96.76, 82.82, and 85.70% for NC vs. AD, MCI vs. NC, and pMCI vs. sMCI, respectively. In addition, the synthetic PET with 32 ROIs features (proposed PET 32) achieves 94.71, 78.42, and 81.05% for NC vs. AD, MCI vs. NC, and pMCI vs. sMCI, respectively. Finally, combining MRI and PET with only 32 ROIs features (proposed MRI-PET 32) achieves 98.24, 94.59, and 87.25% for NC vs. AD, MCI vs. NC, and pMCI vs. sMCI, respectively.

These results verify the effectiveness of the proposed method for discrimination between AD and stages of the disease. Also, these results show the importance of reconstructing the synthetic images with only 32 ROIs features. Moreover, the multimodal data fusion increases the performance of AD diagnosis compared to single modality.

**FIGURE 6:**
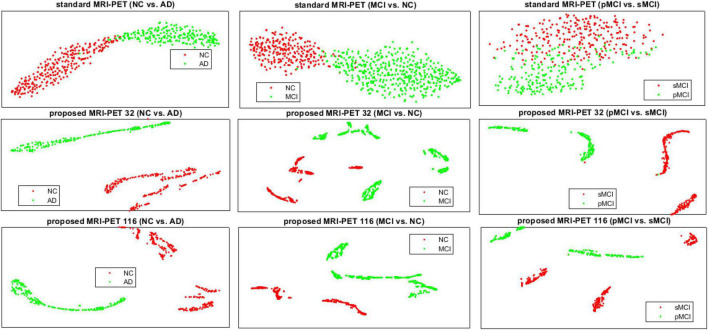
The t-SNE visualization comparison of features between the standard and the proposed method for the three different classification tasks.

In Figure 6, we present the t-SNE feature visualization between the standard and proposed method for NC vs. AD, MCI vs. NC, and pMCI vs. sMCI, respectively. It is easily shown that, our proposed method achieves a better feature discrimination than competing methods. Furthermore, our proposed method has a high intra-class difference for pMCI vs. sMCI. It is noted that, the pMCI vs. sMCI is considered the most challenging classification task since the difference is very subtle. However, our results are better than compared methods (Table 3). This concludes that, our proposed method has the best feature discrimination for different classification tasks.

**FIGURE 7:**
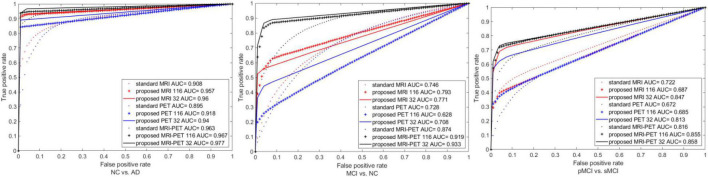
ROC curves comparison between the standard and the proposed method for three the different classification tasks.

We also plot the ROC curves between different configuration of the standard and proposed method as shown in Figure 7. It is clear that, our method has the best area under the ROC curve (AUC) compared to the single modality or combination between modalities. Specifically, the AUC of standard MRI-PET achieves 0.963, 0.874, and 0.816 for NC vs. AD, MCI vs. NC, and pMCI vs. sMCI, respectively. On the other hand, the AUC of the proposed MRI-PET 32 is 0.977, 0.933, and 0.858 for NC vs. AD, MCI vs. NC, and pMCI vs. sMCI, respectively.

### Comparison With Previous Studies

We verify the effectiveness of our method by comparing it many competing methods for NC vs. AD, MCI vs. NC, and pMCI vs. sMCI as shown in Table 4. It is clear that, identifying of the brain disease is increased by our method compared to the recent studies. However, most of recent studies utilized only ROIs instead of utilizing the original images which may induce missing informative features. Furthermore, they mostly used only patches instead of full image for learning their network. Moreover, they often divided the whole learning process into many stages for AD diagnosis.

**TABLE 4:** Algorithm comparisons for the three different classification tasks.

Algorithm	Subject	Modality	NC vs. AD			MCI vs. NC			pMCI vs. sMCI		
			ACC	SEN	SPE	ACC	SEN	SPE	ACC	SEN	SPE
Liu et al., 2014b	198 AD + 229 NC + 225 MCI	MRI	92.0	90.9	93.0	85.3	82.3	88.2	–	–	
Tong et al., 2017	37 AD + 35 NC + 75 MCI	PET + MRI + CSF + genetic	91.4	–	–	77.4	–	–	–	–	–
Zhou et al., 2019a	171 AD + 204 NC + 157 pMCI + 205 sMCI	PET + MRI + SNPs	–	–	–	–	–	–	74.3	–	–
Feng et al., 2019	93 AD + 100 NC + 204 MCI	PET + MRI	94.82	97.70	92.45	–	–	–	–	–	–
Zhou et al., 2019b	190 AD + 226 NC + 389 MCI	PET + MRI + SNPs	91.35	91.75	90.90	–	–	–	–	–	–
Liu et al., 2018b	370 AD + 440 NC + 149 pMCI + 562 sMCI	MRI	90.9	87.9	93.3	–	–	–	73.5	74.4	73.4
Abdelaziz et al., 2021	186 AD + 226 NC + 389 MCI	PET + MRI + SNPs	98.22	97.78	98.76	–	–	–	–	–	–
Suk et al., 2014	93 AD + 101 NC + 76 pMCI + 128 sMCI	MRI	92.38	91.54	94.56	–	–	–	72.42	36.70	90.98
Cheng et al., 2017	199 AD + 229 NC	MRI	86.36	85.93	87.15	–	–	–	–	–	–
Liu et al., 2018a	93 AD + 100 NC + 76 pMCI + 128 sMCI	MRI	92.75	93.48	91.30	–	–	–	76.90	42.11	82.43
Basaia et al., 2019	294 AD + 352 NC + 253 pMCI + 510 sMCI	MRI	99.2	98.9	99.5	–	–	–	75.1	74.8	75.3
Ours	218 AD + 264 NC + 204 pMCI + 273 sMCI	PET + MRI	98.24	98.82	97.52	94.59	90.26	96.98	87.25	94.25	77.89

Hence, in this paper, we develop a technique for utilizing the full neuroimaging data in only one learning process. We integrate the original neuroimaging data with its ROIs features for forcing the network toward the regions with highly related to AD and hence, the early detection of AD improved. Moreover, we evaluate the effectiveness of our method using ADNI-1 and ADNI-2 dataset. Our method achieves the best performance compared to the recent studies in brain disease classification.

### Discussion

In this study, we exhibit the performance of our method *via* three different tasks as shown in Table 4. It is clear that, our method has better performance in most cases. However, the recent studies used ROIs, identified by the handcrafted features through AAL atlas rather than utilizing the original images, which may induce missing informative features. In addition, they trained their network based on the most important patches instead of full images for AD diagnosis in multistage learning scheme. Thus, we integrate the original image features from MRI and PET with ROIs features in one framework. Moreover, we employ the ROIs features for increasing the attention to the highly regions related to AD and hence, the classifier performance improved.

Specifically, we firstly adapt the 116 ROIs features to be suitable for concatenation with the original images by registering every ROI with its corresponding region of the original image and hence, we have one synthetic image for each of neuroimaging subject. Then, we develop a deep learning technique that uses the MRI and PET in only one stage. Also, we learn the original images and synthetic images by applying the CNN to the original images and convolutional auto-encoder to synthetic images such that we combine their features after each convolution layer. Then, the highly learned features from the MRI and PET are combined for classification.

From the experimental results, it is clear that our technique has a superior performance to most of the recent studies in brain disease classification. The primary explanation is that, the proposed technique takes the advantages of the 116 ROIs features and the original images in one framework. Moreover, we apply the synthetic images with only 32 ROIs at the output of the convolutional auto-encoder to force the network to focus on these regions related to AD. This leads to extract the high learned features related to AD after each convolution layer and hence, it improves the diagnosis of AD. Furthermore, we fuse the learned features from multimodal data for classification. This leads to improve the AD diagnosis as shown in Table 4.

The major contributions of our work are as follows. First, we developed a technique that utilizes the original neuroimaging including MRI and PET and their 116 ROIs features in one unified framework. Furthermore, we took the advantages of the 116 ROIs features by converting them to synthetic image by registering every ROI with its corresponding region of the original image. Second, we utilized ADNI-1 and ADNI-2 neuroimaging data for learning the proposed method. Further, Our method has the best performance compared to the competing machine learning and deep learning techniques in brain disease classification. Third, we introduced a deep learning technique that utilizes the multimodal data including MRI and PET in only one stage for brain disease classification.

## Conclusion and Future Work

In this work, we diagnose AD by developing a novel framework that utilized the neuroimaging data (MRI and PET) in one unified framework. Initially, we converted the 116 ROIs features to synthetic images by registering every ROI with its corresponding region of the original image to get one more synthetic image for each modality of every subject. Then, we separately learned each of the neuroimaging and their synthetic images using CNN and convolutional auto-encoder, respectively. In addition, we fused the synthetic features generated from the convolutional auto-encoder with the original image features generated from CNN after each convolution layer to enhance attention to the highly related AD regions. The highly learned features from neuroimaging data were concatenated for identifying the brain disease classification. In this paper, we utilize ADNI-1 and ADNI-2 neuroimaging data for effective training of the proposed method. Experimental results proved the effectiveness of the proposed method compared to the state-of-the-art methods.

Despite our method achieves better performance than most of the recent studies, it still has few limitations First, our technique utilized only the neuroimaging data and ignores the genetic data. However, the combination between neuroimaging and genetic data improves the accuracy of AD diagnosis. Second, we did not consider the estimation of the clinical scores which is considered as one of the important measurements for identifying the patient’s status. Third, we did not consider the relationship among 116 ROIs features during the learning process.

## Data Availability Statement

Publicly available datasets were analyzed in this study. This data can be found here: http://adni.loni.usc.edu.

## Author Contributions

MA: conceptualization, methodology, data processing, software, and writing—review and editing. TW: supervision, guidance, and financial and research support. AE: conceptualization, methodology, visualization, and writing—review and editing. All authors contributed to the article and approved the submitted version.

## Conflict of Interest

The authors declare that the research was conducted in the absence of any commercial or financial relationships that could be construed as a potential conflict of interest.

## Publisher’s Note

All claims expressed in this article are solely those of the authors and do not necessarily represent those of their affiliated organizations, or those of the publisher, the editors and the reviewers. Any product that may be evaluated in this article, or claim that may be made by its manufacturer, is not guaranteed or endorsed by the publisher.

## Footnotes

1
https://adni.loni.usc.edu/
